# Associations between perceived school climate and close friend support among Chinese high school students: a cross-sectional serial indirect-effect analysis of exercise-related basic psychological needs satisfaction and self-esteem

**DOI:** 10.3389/fpsyg.2026.1848230

**Published:** 2026-05-25

**Authors:** Hanyang Cui, Luhui Li, Wenting Liu

**Affiliations:** 1 School of Physical Education, Shihezi University, Shihezi, Xinjiang, China; 2 School of Physical Education, College of Arts and Sciences·Kunming, Kunming, Yunnan, China

**Keywords:** adolescent development, basic psychological needs satisfaction in exercise, Chinese high school students, close friend support, school climate, self-esteem

## Abstract

**Background:**

Close friend support is a key emotional and coping resource during adolescence, yet less is known about the psychological variables that may statistically account for its association with students' perceived school climate.

**Methods:**

We conducted a cross-sectional survey among 1,380 Chinese high school students aged 15–18 years from five senior high schools in Shihezi, Xinjiang, China. Perceived school climate was measured using the 12-item school climate component of the Abbreviated Dual School Climate and School Identification Measure-Student; basic psychological needs satisfaction in exercise, self-esteem, and close friend support were assessed with self-report scales. Covariate-adjusted specific and sequential statistical indirect effects were estimated using PROCESS Model 6 with 5,000 bootstrap resamples while controlling for sex and grade level.

**Results:**

Perceived school climate was positively associated with close friend support (total effect *B* = 0.507, 95% CI [0.463, 0.550]). A direct association remained statistically significant after accounting for both intervening variables (*B* = 0.263, 95% CI [0.217, 0.310]), and the total indirect effect was *B* = 0.243 (95% CI [0.209, 0.279]). Statistically significant indirect effects were observed via basic psychological needs satisfaction in exercise (*B* = 0.084, 95% CI [0.062, 0.107]), via self-esteem (*B* = 0.113, 95% CI [0.088, 0.138]), and sequentially via basic psychological needs satisfaction in exercise and self-esteem (*B* = 0.046, 95% CI [0.035, 0.058]).

**Conclusion:**

A more positive perceived school climate was associated with greater close friend support, with statistical indirect effects via exercise-related basic psychological needs satisfaction and self-esteem. The findings point to practice-relevant considerations for supportive school climates and need-supportive exercise contexts; however, because the data were cross-sectional and adjusted only for sex and grade level, these indirect effects should not be interpreted as evidence of temporal ordering or causal mediation.

## 1 Introduction

Adolescence is a pivotal developmental period marked by rapid changes in social relationships and self-evaluations, during which close peer ties provide essential resources for emotional support, stress buffering, and identity formation. In this study, we focus on close friend support, defined as the perceived exchange of emotional, informational, appraisal, and instrumental support from a close friend. It is treated here as a specific support-based indicator within the broader peer-relationship domain, rather than as a measure of that domain as a whole. Because school is a primary context for “adolescents' daily socialization, students' perceptions” of the school environment may be related to the support they perceive from close friends. School climate refers to students' overall perceptions of school life, including the quality of student-student and student-teacher relationships, academic emphasis, and shared values and collective orientation. Prior research has shown that more positive perceptions of school climate are associated with fewer peer problems and better adjustment (Fredrick et al., 2022). Conversely, negative peer experiences such as exclusion and victimization are linked to poorer subsequent wellbeing (Varela et al., 2021). However, compared with broader indicators of peer functioning, less is known about how students' perceived school climate relates specifically to close friend support and what variables may statistically account for this association among high school students.

Physical education classes and extracurricular exercise may provide school-based interpersonal contexts for understanding how perceived school climate is associated with close friend support, because these settings involve teacher and peer interaction, cooperation, feedback, and experiences relevant to autonomy, competence, and relatedness need satisfaction. Intervention and longitudinal evidence suggests that greater teacher autonomy support in physical education is associated with higher satisfaction of students' basic psychological needs, a more task-involving peer motivational climate, and greater prosocial behavior (Cheon et al., 2019). These findings are consistent with the possibility that need-supportive exercise experiences may be associated with broader peer interaction patterns and, in turn, with close friend support. In addition, self-esteem is a key psychological resource for adolescents' relational engagement and social adaptation. Sociometer Theory proposes that self-esteem functions as a subjective gauge of perceived acceptance and relational value, shaping interpersonal security and how individuals express and maintain close relationships (Leary and Baumeister, 2000). Building on these theoretical and empirical accounts, the present study examined whether students' perceived school climate was associated with close friend support among Chinese high school students and whether basic psychological needs satisfaction in exercise and self-esteem statistically accounted for this association independently and sequentially. Specifically, this study aimed to (1) characterize the association between perceived school climate and close friend support; and (2) examine whether basic psychological needs satisfaction in exercise and self-esteem statistically accounted for this association, independently and sequentially. Beyond these empirical aims, the findings may provide practice-relevant considerations for coordinated efforts in school climate improvement, physical education, and school-based mental health education.

## 2 Literature review and research hypotheses

### 2.1 School climate and close friend support

School climate reflects students' overall perceptions of the school environment. In the present study, perceived school climate refers to students' global perceptions of school life, emphasizing peer and teacher-student relationships, academic emphasis, shared values, and a sense of common orientation. Accordingly, our focus is on students' day-to-day school experiences rather than objective ecological indicators. A systematic review suggested that schools are a key setting for interventions aimed at supportive peer interactions, with identity-related processes and socio-emotional competencies occupying central explanatory roles (Mitic et al., 2021). Notably, much prior work has operationalized peer-related outcomes using indicators such as peer problems, bullying experiences, school connectedness/satisfaction, or wellbeing. Although these indicators are not equivalent to close friend support, they provide indirect evidence that perceived school climate is associated with peer-related functioning and the interpersonal conditions under which supportive exchanges with close friends may occur. Empirical studies have shown that more favorable perceived school safety and order are associated with fewer peer problems (Fredrick et al., 2022), and that climate dimensions such as anti-bullying norms and respect for diversity are linked to better subjective wellbeing (Hatzichristou et al., 2020). Related evidence further highlights peer interaction processes: perceived school climate is associated with bullying experiences, and bullying has been reported as an intermediary factor linking school climate to other behavioral risks (Dogan and Demircioglu, 2025); multidimensional school climate has also been linked to bullying perpetration indirectly through victimization, moral disengagement, and empathy (Montero-Carretero et al., 2021). Longitudinal findings similarly indicate that school connectedness and satisfaction are related to bullying experiences and that bullying victimization predicts subsequent lower life satisfaction (Varela et al., 2021). Taken together, a more positive perceived school climate may be associated with a more predictable, respectful, and supportive interpersonal context that is conducive to supportive exchanges within close friendships. Therefore, we propose Hypothesis 1: Students' perceived school climate is positively associated with close friend support.

### 2.2 Indirect role of basic psychological needs satisfaction in exercise

Basic psychological needs satisfaction in exercise (autonomy, competence, and relatedness) is a core construct in self-determination theory and basic psychological needs theory for understanding adolescents' motivation and adjustment in physical activity contexts. Drawing on the hierarchical model of intrinsic and extrinsic motivation, physical education classes and extracurricular exercise can be understood as school-based interpersonal settings in which students who perceive a more supportive and well-structured school climate may also report more organized, cooperative, and need-supportive exercise experiences, including stronger perceptions of autonomy, competence, and relatedness during exercise. These motivational experiences may be associated with broader peer-related functioning, including greater engagement, cooperative interaction patterns, and prosocial orientations that are conducive to supportive exchanges within close friendships. Importantly, most evidence for the “contextual support → needs satisfaction → social outcomes” pathway has been drawn from physical education-level contextual variables such as teacher autonomy support, motivational climate, or instructional models. Although this evidence does not directly test general perceived school climate or close friend support, it supports the plausibility that school-based exercise experiences may be relevant to peer-related social functioning. Consistent with this logic, intervention and multilevel studies have shown that increasing physical education teachers' autonomy support enhances students' needs satisfaction, fosters a more task-involving peer climate, increases prosocial behavior, and reduces antisocial behavior (Cheon et al., 2018, 2019). Cross-sectional findings likewise suggest that teacher autonomy support is indirectly associated with lower bullying perpetration and victimization through a sequential pathway involving needs satisfaction and self-determined motivation (Montero-Carretero et al., 2020), further illustrating how need satisfaction in physical education may be linked to peer-related outcomes. Evidence on skill transfer further indicates that needs satisfaction has been reported to mediate the association between teacher support and life skills and to predict subsequent life-skill development (Cronin et al., 2019, 2020; Ji et al., 2025), including teamwork and social communication skills that are behaviorally relevant to supportive exchanges in close friendships. Higher-level syntheses also suggest that, relative to more traditional instruction, cooperative and supportive physical education models are generally more conducive to needs satisfaction and prosocial attitudes (Manninen and Campbell, 2022). Although much of this literature has emphasized life skills, prosociality, or bullying rather than close friend support *per se*, evidence from a Chinese high-school sample indicates that peer support is associated with relatedness need satisfaction and social skills (Primo et al., 2023), providing more proximal support for the view that exercise-related needs satisfaction may be associated with close friend support. Therefore, we propose Hypothesis 2: More positive perceived school climate is expected to be associated with greater close friend support indirectly through higher basic psychological needs satisfaction in exercise.

### 2.3 Indirect role of self-esteem

Self-esteem is a core indicator of adolescents' self-worth and self-acceptance and an important psychological resource for relational engagement and social adjustment. Sociometer Theory conceptualizes self-esteem as an internal monitoring system of perceived acceptance and relational value: cues of respect, inclusion, and rejection in interpersonal contexts are reflected in self-esteem and, in turn, may shape how individuals approach, express, and maintain relationships (Leary and Baumeister, 2000). From a school-context perspective, a more positive perceived school climate-characterized by respectful peer and teacher-student relationships, clearer norms, and a more supportive interpersonal tone-may provide more stable acceptance-related cues and be associated with higher self-esteem. Empirical research has reported stable associations between school engagement/connectedness and self-esteem, including reciprocal relations over time (Karababa, 2020), and self-esteem has been examined as a pathway linking school engagement with psychological outcomes (Bang et al., 2020), highlighting its relevance in connecting school-related experiences with adjustment. At the same time, evidence on directionality is not uniform: some studies suggest that self-esteem more robustly predicts later school belonging, whereas the reverse effect is less stable (Hernández et al., 2017; Perry and Lavins-Merillat, 2018), warranting caution in interpreting causal ordering. With respect to interpersonal outcomes, meta-analytic evidence indicates that self-esteem is positively associated with multiple interpersonal indicators, and longitudinal subsets support a prospective association of self-esteem with later interpersonal experiences and relationship outcomes (Cameron and Granger, 2019). Although friendship quality and broader interpersonal outcomes are not identical to close friend support, they provide related evidence that self-esteem is linked to adolescents' close relational experiences. For example, a systematic review of friendship quality and adolescents' subjective wellbeing reported a positive association between friendship quality and self-esteem in five of six applicable studies (Alsarrani et al., 2022), and friend support and self-esteem remain related yet partially distinct across sources of support (Laursen et al., 2021). Within school peer ecologies, exclusion and rejection are associated with lower self-esteem (Danneel et al., 2019), and social exclusion can be linked to subsequent adjustment via self-esteem and related pathways (Arslan, 2019), supporting the plausibility of an indirect association in which school-context cues relate to close friend support through self-esteem. Therefore, we propose Hypothesis 3: More positive perceived school climate is expected to be associated with greater close friend support indirectly through higher self-esteem.

### 2.4 Sequential indirect association of basic psychological needs satisfaction in exercise and self-esteem

The association between students' perceived school climate and close friend support may reflect not only direct interpersonal conditions but also a hypothesized sequential association in which exercise-related motivational experiences are linked to self-evaluative processes relevant to close relationships. Self-determination theory emphasizes that satisfaction of autonomy, competence, and relatedness needs supports more stable positive functioning and adaptive behavior (Ryan and Deci, 2000). In physical activity settings, experiences of meaningful choice, skill improvement, and connection with others may provide competence- and connection-related cues that are associated with more positive self-evaluations and higher self-esteem. For example, longitudinal work in adolescent sport teams has linked contextual support with competence and relatedness need satisfaction and with higher global self-esteem via competence-related experiences (Coatsworth and Conroy, 2009). A systematic review and meta-analysis of physical activity interventions also indicates a consistent association between exercise participation and improvements in self-esteem (Liu et al., 2015).

In relation to close friend support, Sociometer Theory frames self-esteem as an internal gauge of social acceptance and relational value that may shape sensitivity to rejection cues and patterns of relational investment (Leary and Baumeister, 2000). Reviews of longitudinal evidence indicate that high self-esteem prospectively predicts success and wellbeing in important life domains, including relationships (Orth and Robins, 2014), and friend support and self-esteem are systematically related among adolescents (Rueger et al., 2010). Integrating these perspectives, a more positive perceived school climate may be associated with higher needs satisfaction in exercise, which may relate to higher self-esteem and, in turn, to greater close friend support. Therefore, we propose Hypothesis 4: More positive perceived school climate is expected to be associated with greater close friend support via a sequential indirect association involving higher basic psychological needs satisfaction in exercise and higher self-esteem.

**Figure 1:**
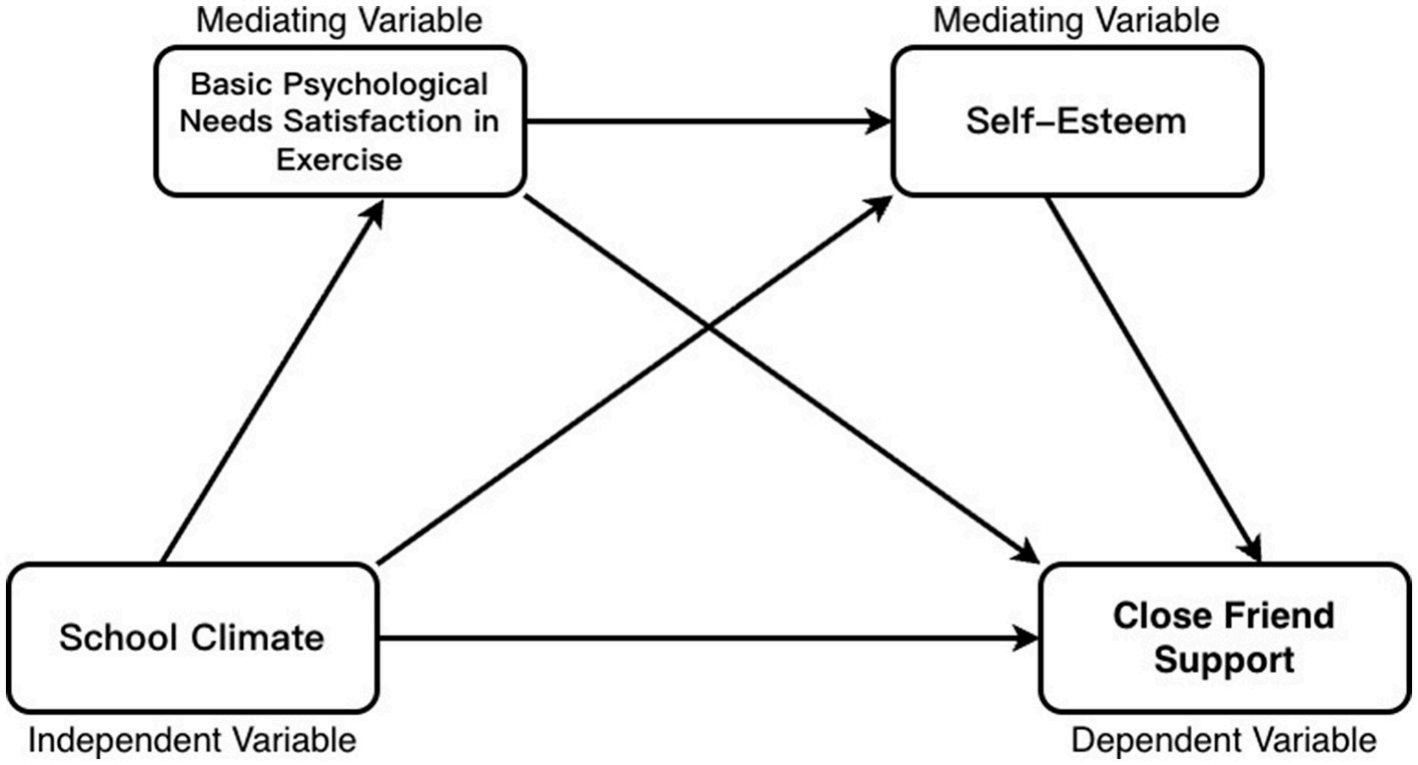
Hypothesized statistical model of the association between perceived school climate and close friend support via basic psychological needs satisfaction in exercise and self-esteem.

**Figure 2:**
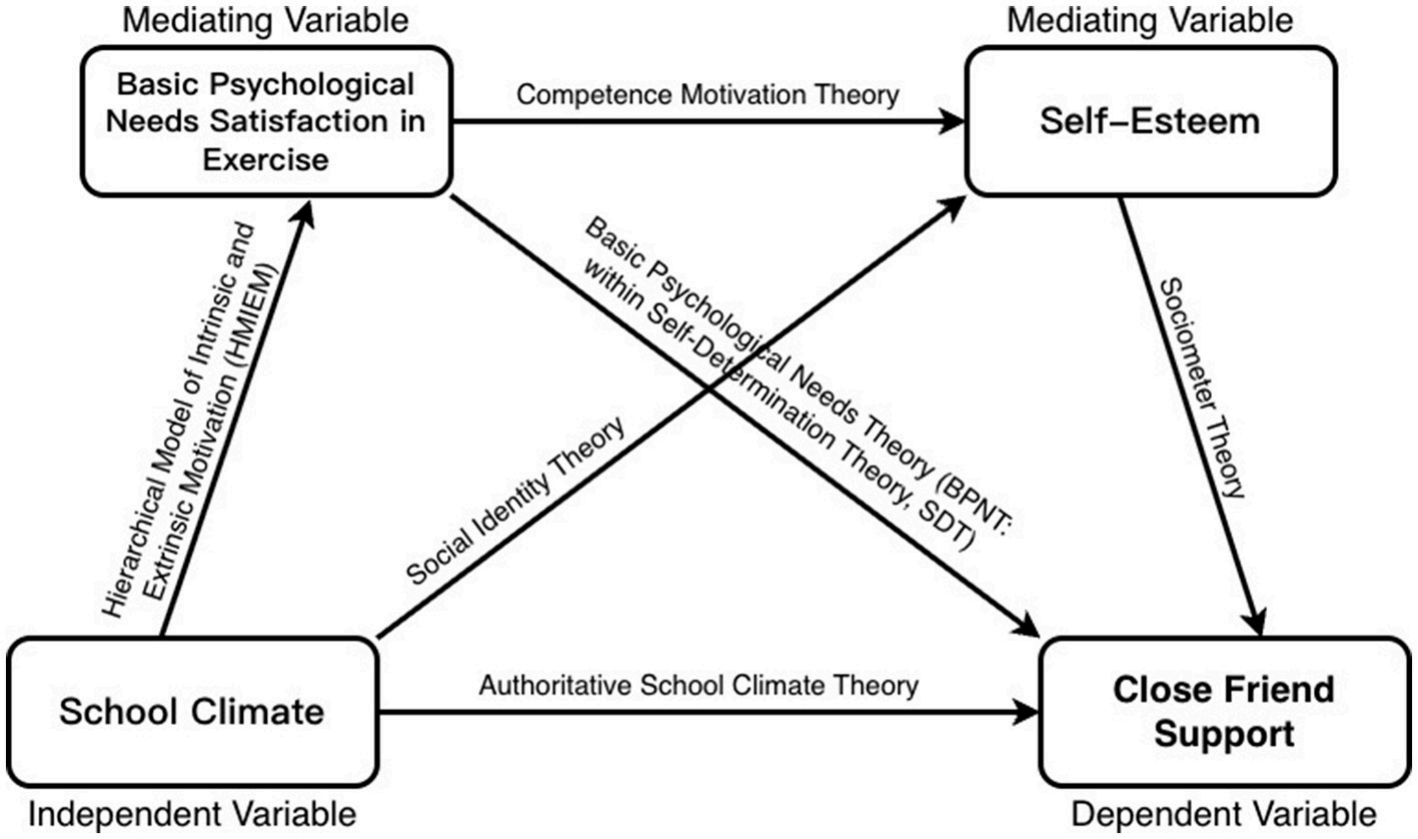
Conceptual rationale for the proposed associations among perceived school climate, basic psychological needs satisfaction in exercise, self-esteem, and close friend support.

Taken together, prior research provides converging support from multiple angles, including school-context relational and normative cues, motivational experiences in exercise, and the role of self-esteem in interpersonal functioning, supporting the plausibility that students' perceived school climate may be linked to close friend support through exercise-related needs satisfaction and self-esteem. However, studies that integrate perceived school climate, basic psychological needs satisfaction in exercise, and self-esteem within a single model-and evaluate their sequential indirect association using close friend support as a proximal relational outcome-remain relatively limited. Given the cross-sectional design, the proposed sequence is treated as a theory-informed statistical ordering rather than evidence of temporal or causal ordering. Accordingly, Figure 1 presents the hypothesized statistical model, and Figure 2 summarizes the conceptual rationale for the proposed associations. Based on this framework, the following hypotheses were formulated: Hypothesis 1 examines the association between students' perceived school climate and close friend support; Hypothesis 2 examines the indirect association through basic psychological needs satisfaction in exercise; Hypothesis 3 examines the indirect association through self-esteem; and Hypothesis 4 examines the sequential indirect association through basic psychological needs satisfaction in exercise and self-esteem.

## 3 Materials and methods

### 3.1 Participants

Using a stratified cluster sampling design, we conducted an electronically administered questionnaire survey in five senior high schools in Shihezi, Xinjiang, China, from March 10 to 20, 2026 (all schools offered only the senior high level and did not enroll junior high students). Eligible participants were enrolled senior high school students in Shihezi aged 15–18 years. Grade level (Grade 10, Grade 11, and Grade 12) served as the stratification variable; within each grade, intact classes were randomly selected, and the electronic questionnaire was administered collectively during class time with standardized instructions provided by trained undergraduate research assistants to ensure procedural consistency. A total of 1,446 questionnaires were distributed; after predefined data screening, 1,380 valid questionnaires were retained (valid response rate = 95.44%). Participants were 15–18 years old, including 692 male and 688 female students; 563 were in Grade 10, 491 in Grade 11, and 326 in Grade 12. Before analysis, questionnaires were screened according to predefined criteria. Responses were excluded if they had a completion time of < 5 min, more than 20% missing responses, or obvious logical inconsistencies. These criteria were applied to reduce the influence of insufficiently engaged or incomplete responses. Following school-wide notification, participants received a brief description of the study purpose and procedures before entering the formal questionnaire. For students younger than 18 years, written informed consent was obtained from a parent or legal guardian, and student assent was obtained; students aged 18 years provided their own written informed consent. Only students who agreed to participate were allowed to proceed, and participants were informed that participation was voluntary, responses were anonymous and confidential, and results would be used for research purposes only. The study protocol and informed consent form were reviewed and approved by the Science and Technology Ethics Committee of the First Affiliated Hospital of Shihezi University (IRB No.: KJ2026-063-01). All procedures were conducted in accordance with the approved protocol, the ethical standards of the approving committee, and the Declaration of Helsinki.

### 3.2 Measures

#### 3.2.1 Abbreviated dual school climate and school identification measure-student

Perceived school climate was assessed using the Chinese version of the Abbreviated Dual School Climate and School Identification Measure-Student (SCASIM-St15; Lee et al., 2017; Gálvez-Nieto et al., 2021; Yu et al., 2022). The original instrument includes 15 items comprising four school climate domains (student-student relations, student-staff relations, academic emphasis, and shared values and approach) and one school identification domain. In the present study, perceived school climate was operationalized using only the 12 school climate items, and the three school identification items were excluded from scoring and were not included in the subsequent analyses. All items were rated on a 5-point Likert scale (1 = strongly disagree, 5 = strongly agree). Domain scores were computed as mean item scores, and the school climate composite score was calculated as the mean of the 12 school climate items, with higher scores indicating a more positive overall appraisal of the school climate. In the present sample, Cronbach's alpha coefficients for the four school climate domains ranged from 0.840 to 0.859, and confirmatory factor analysis showed good fit (χ^2^/df = 1.392, RMSEA = 0.017, CFI = 0.998, TLI = 0.997).

#### 3.2.2 Psychological need satisfaction in exercise scale

Basic psychological needs satisfaction in exercise was assessed using the Chinese adolescent version of the Psychological Need Satisfaction in Exercise Scale (Wilson et al., 2006; Wang and Yu, 2019). The scale assessed autonomy, competence, and relatedness and included 12 items (e.g., “I can decide what kind of exercise I want to do.”) rated on a 5-point Likert scale (1 = strongly disagree, 5 = strongly agree). Subscale scores were computed as mean item scores; consistent with the study model, the mean of all 12 items was used as the composite indicator of basic psychological needs satisfaction in exercise (BPNSE), with higher scores reflecting greater needs satisfaction in the exercise context. In the present sample, Cronbach's alpha coefficients for autonomy, competence, and relatedness were 0.876, 0.879, and 0.877, respectively, and confirmatory factor analysis indicated good fit (χ^2^/df = 1.822, RMSEA = 0.024, CFI = 0.996, TLI = 0.994).

#### 3.2.3 Rosenberg self-esteem scale

Self-esteem was assessed using the Rosenberg Self-Esteem Scale (RSES-10; Rosenberg, 1965; Tian, 2022). The instrument was unidimensional and included 10 items, five of which were reverse-worded (e.g., “I don't think I have much respect for myself.”). To harmonize response formats across measures, items were rated on a 5-point Likert scale (1 = strongly disagree, 5 = strongly agree) by adapting the number of response options without changing item meaning or scoring direction (Preston and Colman, 2000). Reverse-worded items were recoded before aggregation, and the mean of the 10 items was calculated as the self-esteem score (SE), with higher scores indicating higher self-esteem. In the present sample, Cronbach's alpha was 0.948, and confirmatory factor analysis showed good fit (χ^2^/df = 2.462, RMSEA = 0.033, CFI = 0.995, TLI = 0.994).

#### 3.2.4 Close friend subscale of the child and adolescent social support scale

Close friend support was assessed using the close friend subscale (12 items) of the Child and Adolescent Social Support Scale (CASSS-Close Friend 12; Malecki and Demaray, 2002; Luo et al., 2017). The subscale covers four types of support-emotional, informational, appraisal, and instrumental-and includes 12 items (e.g., “My close friend sticks up for me if others are treating me badly.”). The frequency-of-support response format was used, and the original 6-point frequency scale was adapted to a 5-point scale (1 = never, 5 = always) while maintaining the original meaning (Preston and Colman, 2000). Support-domain scores were computed as mean item scores, and the mean of all 12 items was used to operationalize close friend support as the study outcome, with higher scores indicating greater perceived support from close friends. In the present sample, Cronbach's alpha coefficients for the four support domains were 0.855, 0.834, 0.860, and 0.845, respectively, and confirmatory factor analysis indicated good fit (χ^2^/df = 1.424, RMSEA = 0.018, CFI = 0.998, TLI = 0.997).

### 3.3 Statistical analysis

Data were processed and analyzed using SPSS 26.0 and AMOS 26.0. Means and standard deviations were computed for all variables, common method variance was preliminarily examined using Harman's single-factor test, and internal consistency was evaluated using Cronbach's alpha. School climate, basic psychological needs satisfaction in exercise, self-esteem, and close friend support were operationalized as observed variables using the mean scores across all items of their respective scales and were entered into the subsequent correlation, regression, and indirect-effect analyses. Because PROCESS estimates observed-variable models, composite mean scores were used after acceptable reliability and factorial validity had been established. For consistency with the tables and figures, these variables are abbreviated as school climate (SC), basic psychological needs satisfaction in exercise (BPNSE), self-esteem (SE), and close friend support (CFS). A four-construct confirmatory factor analysis was conducted in AMOS to examine the measurement model, and model fit was evaluated using χ^2^/df, RMSEA, CFI, and TLI. Differences by sex were tested using independent-samples *t* tests, and differences by grade level and age were examined using one-way analysis of variance (ANOVA). For age-group comparisons, age was categorized by completed years. Because age was closely aligned with grade level in this senior-high-school sample, grade level rather than age was retained as the educational-stage covariate in the indirect-effect models to avoid redundant adjustment. Sex was also included as a covariate because it is a relevant demographic variable and sex differences were observed in the study variables. Detailed indicators of socioeconomic status, such as parental education, parental occupation, or household income, as well as family support, academic stress, psychological distress, pre-existing friendship quality, peer network characteristics, and exercise participation characteristics, such as type, frequency, and intensity, were not available in the present dataset and therefore could not be included as covariates. Accordingly, the indirect-effect models were interpreted as covariate-adjusted cross-sectional association models rather than as fully confounding-adjusted explanatory models. The primary PROCESS analyses were conducted at the individual-student level; possible non-independence due to class- or school-level clustering was not explicitly modeled and is addressed as a limitation. Associations among variables were examined using Pearson correlation analyses. Statistical indirect effects were estimated using Hayes' PROCESS macro (Model 6), in which two specific indirect effects (school climate → basic psychological needs satisfaction in exercise → close friend support; school climate → self-esteem → close friend support) and the sequential indirect effect (school climate → basic psychological needs satisfaction in exercise → self-esteem → close friend support) were estimated in the same model while controlling for sex and grade level. Bootstrap confidence intervals based on 5,000 resamples were used to estimate 95% confidence intervals, and an indirect effect was considered statistically significant when the 95% confidence interval did not include zero. All tests were two-tailed, with the significance level set at p < 0.05. Given the cross-sectional design, the model was used to estimate statistical sequential indirect effects, which are interpreted in this study as statistical associations consistent with the hypothesized ordering rather than as evidence of temporal ordering or causal mediation.

## 4 Results

### 4.1 Descriptive statistics for school climate, basic psychological needs satisfaction in exercise, self-esteem, and close friend support

Table 1 presents the descriptive statistics and group comparisons for school climate (SC), basic psychological needs satisfaction in exercise (BPNSE), self-esteem (SE), and close friend support (CFS). In the full sample, the mean scores were SC (M = 3.60, SD = 0.87), BPNSE (M = 3.68, SD = 0.87), SE (M = 3.60, SD = 1.01), and CFS (M = 3.73, SD = 0.85). Sex differences were statistically significant for all four variables (*t* = 7.169–8.857, all *p* < 0.001), with female students scoring higher than male students on SC, BPNSE, SE, and CFS. One-way analyses of variance showed significant grade-level differences across the four variables (*F* = 29.425–49.537, all *p* < 0.001), and age-group comparisons also indicated significant differences (*F* = 18.786–33.263, all *p* < 0.001).

**Table 1:** Descriptive statistics (M ± SD) and group difference tests for SC, BPNSE, SE, and CFS.

Group	N	School climate (SC)	Basic psychological needs satisfaction in exercise (BPNSE)	Self-esteem (SE)	Close friend support (CFS)
Male	692	3.44 ± 0.86	3.48 ± 0.86	3.37 ± 1.00	3.55 ± 0.84
Female	688	3.77 ± 0.85	3.88 ± 0.82	3.83 ± 0.98	3.90 ± 0.82
Overall	1380	3.60 ± 0.87	3.68 ± 0.87	3.60 ± 1.01	3.73 ± 0.85
Sex differences (t)		7.169^***^	8.857^***^	8.754^***^	7.861^***^
Grade differences (F)		29.425^***^	30.672^***^	46.005^***^	49.537^***^
Age differences (F)		33.263^***^	21.764^***^	24.397^***^	18.786^***^

SC, perceived school climate; BPNSE, basic psychological needs satisfaction in exercise; SE, self-esteem; and CFS, close friend support. Values are presented as M ± SD. Sex differences were tested using independent-samples *t* tests; grade and age differences were tested using one-way ANOVAs. *t* values are reported as absolute values. ^***^ *p* < 0.001.

### 4.2 Preliminary examination of common method variance

Common method variance was preliminarily examined using Harman's single-factor test. All measurement items from the four scales were entered simultaneously into an unrotated principal component analysis. A total of 12 components with eigenvalues > 1.0 were extracted, and the first component accounted for 35.15% of the total variance, which was below the 40% threshold commonly used in Harman's single-factor test (Chen et al., 2025). These results did not indicate a dominant single factor; however, this limited diagnostic cannot rule out common method variance.

### 4.3 Correlation analysis of school climate, basic psychological needs satisfaction in exercise, self-esteem, and close friend support

As shown in Table 2, school climate (SC) was positively correlated with basic psychological needs satisfaction in exercise (BPNSE) (*r* = 0.480, *p* < 0.001), self-esteem (SE) (*r* = 0.552, *p* < 0.001), and close friend support (CFS) (*r* = 0.565, *p* < 0.001). BPNSE was positively correlated with SE (*r* = 0.545, *p* < 0.001) and CFS (*r* = 0.528, *p* < 0.001), and SE was positively correlated with CFS (*r* = 0.607, *p* < 0.001). The zero-order positive association between SC and CFS was consistent with Hypothesis 1 and provided a correlational basis for the subsequent indirect-effect analyses.

**Table 2:** Means, standard deviations, and correlations among study variables.

Variable	M	SD	1	2	3	4
School climate (SC)	3.60	0.87	1			
Basic psychological needs satisfaction in exercise (BPNSE)	3.68	0.87	0.480^***^	1		
Self-esteem (SE)	3.60	1.01	0.552^***^	0.545^***^	1	
Close friend support (CFS)	3.73	0.85	0.565^***^	0.528^***^	0.607^***^	1

SC, perceived school climate; BPNSE, basic psychological needs satisfaction in exercise; SE, self-esteem; and CFS, close friend support. *N* = 1,380. Pearson correlations (two-tailed) are reported. ^***^ *p* < 0.001.

### 4.4 Testing statistical indirect effects of basic psychological needs satisfaction in exercise and self-esteem in the association between perceived school climate and close friend support

**Figure 3:**
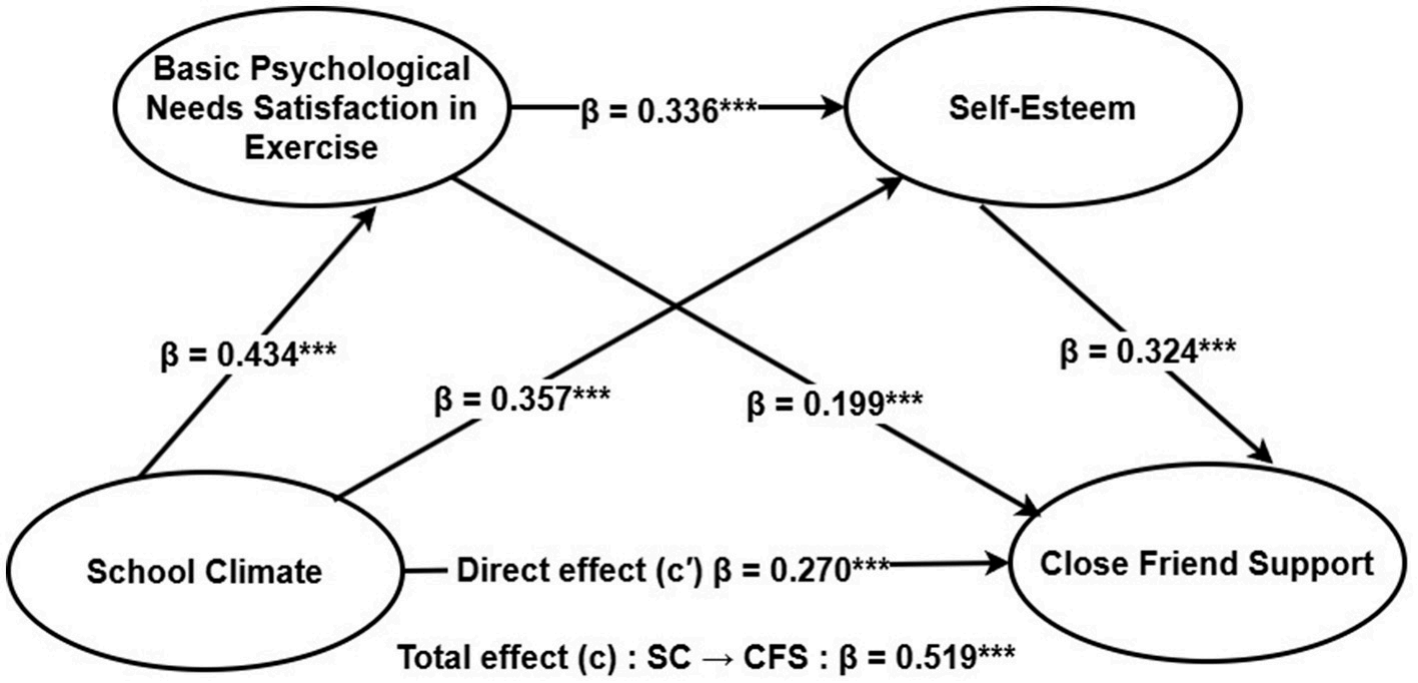
Tested statistical model with standardized path coefficients. Standardized coefficients (β) are shown. Sex and grade level were included as covariates. The unstandardized total, direct, and indirect effects and bootstrap confidence intervals are reported in Table 4. *N* = 1,380. ^***^p < 0.001.

To examine covariate-adjusted statistical indirect effects in the association between perceived school climate and close friend support, analyses were conducted using Hayes' PROCESS macro (Model 6) with 5,000 bootstrap resamples while controlling for sex and grade level. Standardized path coefficients (β) are reported in Table 3 to facilitate comparisons of relative path coefficients, whereas Figure 3 provides a visual summary of the tested model. The total effect, direct effect, and specific indirect effects are reported as unstandardized effects (B) in Table 4 and evaluated using bootstrap 95% confidence intervals (CIs). In addition, confirmatory factor analysis of the four-construct measurement model was performed in AMOS 26.0, and the model fit was acceptable (χ^2^/df = 1.468, RMSEA = 0.018, CFI = 0.988, TLI = 0.988).

**Table 3:** Regression analysis of the tested indirect-effect model.

Variable	Basic psychological needs satisfaction in exercise		Self-esteem		Close friend support		Total-effect model	
	β	*t*	β	*t*	β	*t*	β	*t*
School climate	0.434^***^	18.059	0.357^***^	15.096	0.270^***^	11.147	0.519^***^	23.036
Basic psychological needs satisfaction in exercise	—	—	0.336^***^	14.100	0.199^***^	8.224	—	—
Self-esteem	—	—	—	—	0.324^***^	12.679	—	—
*R* ^2^	0.261		0.422		0.480		0.349	
*F*	162.084^***^		250.775^***^		254.037^***^		246.340^***^	

SC, perceived school climate; BPNSE, basic psychological needs satisfaction in exercise; SE, self-esteem; and CFS, close friend support. Standardized coefficients (β) are reported. Sex and grade level were included as covariates in all models. ^***^ *p* < 0.001. Unstandardized estimates (B) for the total, direct, and indirect effects are reported in Table 4.

**Table 4:** Bootstrap estimates of direct, total, and statistical indirect effects.

Path	Unstandardized effect (B)	Proportion of total effect	95% CI	
			LL	UL
SC → BPNSE → CFS	0.084	16.62%	0.062	0.107
SC → SE → CFS	0.113	22.29%	0.088	0.138
SC → BPNSE → SE → CFS	0.046	9.12%	0.035	0.058
Total indirect	0.243	48.03%	0.209	0.279
Direct effect (c')	0.263	—	0.217	0.310
Total effect (c)	0.507	—	0.463	0.550

SC, perceived school climate; BPNSE, basic psychological needs satisfaction in exercise; SE, self-esteem; and CFS, close friend support. Effects are unstandardized estimates (B) and are reported with percentile bootstrap 95% confidence intervals based on 5,000 resamples from PROCESS Model 6. Sex and grade level were included as covariates. Proportions are computed relative to the unstandardized total effect (c). Standardized path coefficients (β) are reported in Table 3 and Figure 3. Because the data were cross-sectional, indirect effects should be interpreted as statistical indirect associations rather than evidence of temporal ordering or causal mediation. *N* = 1,380. Minor discrepancies are due to rounding.

Perceived school climate was positively associated with basic psychological needs satisfaction in exercise (β = 0.434, *t* = 18.059, *p* < 0.001, *R*^2^ = 0.261). When school climate and basic psychological needs satisfaction in exercise were entered simultaneously, both school climate (β = 0.357, *t* = 15.096, *p* < 0.001) and basic psychological needs satisfaction in exercise (β = 0.336, *t* = 14.100, *p* < 0.001) were positively associated with self-esteem (*R*^2^ = 0.422). When school climate, basic psychological needs satisfaction in exercise, and self-esteem were entered simultaneously, all three variables were positively associated with close friend support (school climate: β = 0.270, *t* = 11.147, *p* < 0.001; basic psychological needs satisfaction in exercise: β = 0.199, *t* = 8.224, *p* < 0.001; self-esteem: β = 0.324, *t* = 12.679, *p* < 0.001), with an explained variance of *R*^2^ = 0.480. In the total-effect model, school climate was positively associated with close friend support (β = 0.519, *t* = 23.036, *p* < 0.001, *R*^2^ = 0.349).

Bootstrap-based indirect-effect results are shown in Table 4. The total effect of school climate on close friend support was significant (*B* = 0.507, 95% CI [0.463, 0.550]). After including basic psychological needs satisfaction in exercise and self-esteem, the direct effect of school climate remained significant (*B* = 0.263, 95% CI [0.217, 0.310]), and the total indirect effect was *B* = 0.243 (95% CI [0.209, 0.279]), accounting for 48.03% of the total effect. The bootstrap 95% CIs for the three specific indirect pathways did not include zero: SC → BPNSE → CFS (*B* = 0.084, 95% CI [0.062, 0.107]), SC → SE → CFS (*B* = 0.113, 95% CI [0.088, 0.138]), and SC → BPNSE → SE → CFS (*B* = 0.046, 95% CI [0.035, 0.058]). These results were consistent with Hypotheses 2–4 and indicated a pattern in which both the direct association and the statistical indirect associations remained evident. Given the cross-sectional nature of the data, these indirect effects should be interpreted as statistical associations consistent with the hypothesized ordering rather than as evidence of temporal ordering or causal mediation.

## 5 Discussion

This study examined whether basic psychological needs satisfaction in exercise and self-esteem statistically accounted for the association between students' perceived school climate and close friend support among Chinese high school students. Overall, the findings indicated a pattern in which both the direct association and the statistical indirect associations remained evident. The total indirect effect represented 48.03% of the total effect of perceived school climate on close friend support, with the remaining 51.97% represented by the direct association. In terms of the relative contributions of the statistical indirect associations, the association through basic psychological needs satisfaction in exercise represented 16.62% of the total effect, the association through self-esteem represented 22.29%, and the sequential indirect association through basic psychological needs satisfaction in exercise and self-esteem represented 9.12%. Together, these findings indicate that the association between perceived school climate and close friend support can be statistically decomposed into direct and indirect components involving exercise-related need satisfaction and self-esteem. Because the indirect-effect models adjusted only for sex and grade level, these findings should not be interpreted as fully confounding-adjusted estimates or as evidence of a highly specific explanatory pathway. Given the cross-sectional design, they should be interpreted as statistical associations consistent with the hypothesized order rather than as evidence of temporal ordering or causal mediation.

### 5.1 School climate and close friend support

Correlation analyses indicated that students' perceived school climate was positively associated with close friend support (*r* = 0.565, *p* < 0.001). After controlling for sex and grade level, the total effect of perceived school climate on close friend support was significant (*B* = 0.507, 95% CI [0.463, 0.550]), and the direct effect remained significant after including both intervening variables (*B* = 0.263, 95% CI [0.217, 0.310]). Together, these findings indicate a pattern in which perceived school climate was associated with close friend support both through the statistical indirect associations examined in this study and through a remaining direct association not accounted for by basic psychological needs satisfaction in exercise and self-esteem.

It is important to note that the outcome examined here was close friend support, a specific support-based aspect of the broader peer-relationship domain, rather than that domain as a whole. The broader literature has reported consistent associations between school climate and adolescents' interpersonal and adjustment outcomes, particularly for dimensions related to relational quality, safety, and fairness (Wang and Degol, 2016; Cornell et al., 2016; Long et al., 2021). Conceptually, from an authoritative school climate perspective, a school environment characterized by clear and consistently enforced rules alongside supportive adult-student relationships may reduce uncertainty in peer interactions and create conditions that are conducive to trust-building and supportive exchanges among close friends. Empirical work is generally consistent with this account: teacher and peer support have been linked to higher perceived safety and fairness in contexts of peer victimization (Coyle et al., 2022), and positive school climate has been associated with lower aggression through improved self-control (Li et al., 2021) as well as higher prosocial behavior partly via social-emotional competence (Pan et al., 2023). Longitudinal evidence also suggests that school climate is prospectively associated with prosocial tendencies through protective psychological resources such as resilience (Camacho et al., 2025). In the Chinese senior-high-school context, students' peer interactions are often embedded in relatively stable class groups, strong academic demands, teacher-led school management, and collective school norms, which may shape how students perceive school climate and how opportunities for supportive close-friend interactions emerge. In the context of the present cross-sectional design, the observed associations should be interpreted as statistical patterns rather than evidence of temporal ordering or causality. From a practice-relevant perspective, these findings point to the potential relevance of high support-high structure school environments, including transparent rules, teacher support, anti-bullying efforts, and peer-help programs, for conditions associated with supportive peer interactions. Future research should use longitudinal, multi-informant, and multilevel designs to examine school-level effects and their temporal dynamics more rigorously.

### 5.2 Indirect role of basic psychological needs satisfaction in exercise

The findings were consistent with Hypothesis 2, indicating a statistically significant indirect association through basic psychological needs satisfaction in exercise. The specific indirect effect through basic psychological needs satisfaction in exercise was *B* = 0.084, with a bootstrap 95% CI of [0.062, 0.107]. At the path level, perceived school climate was positively associated with basic psychological needs satisfaction in exercise (β = 0.434, *p* < 0.001), and basic psychological needs satisfaction in exercise was positively associated with close friend support (β = 0.199, *p* < 0.001).

This pattern is consistent with evidence from physical education and exercise settings suggesting an association pattern linking supportive contexts, need satisfaction, and social functioning. Autonomy-supportive teaching and supportive peer climates in physical education have been associated with greater need satisfaction and more adaptive social functioning (Vasconcellos et al., 2020; Cheon et al., 2023), and mastery-oriented climates created by teachers and peers have been linked to autonomy, competence, and relatedness satisfaction (Rodrigues et al., 2020; Langøy et al., 2024). Related evidence also suggests that acceptance experiences in physical education are associated with children's social competence via peer-related need satisfaction (de Bruijn and Grimminger-Seidensticker, 2025), and school-based need satisfaction has been linked longitudinally to prosocial behavior (Tian et al., 2018). In team sport contexts, need satisfaction has similarly been identified as an important correlate in the association between autonomy-supportive climates and prosocial behavior (Hodge and Gucciardi, 2015). Although the present study did not directly assess physical education instructional practices or peer motivational climates, and although much of this literature concerns broader social functioning rather than close friend support specifically, the results are statistically consistent with the possibility that exercise-related need satisfaction is associated with supportive friendship exchanges.

### 5.3 Indirect role of self-esteem

The findings were also consistent with Hypothesis 3, indicating a statistically significant indirect association through self-esteem. After controlling for sex and grade level and accounting for basic psychological needs satisfaction in exercise, perceived school climate remained positively associated with self-esteem (β = 0.357, *p* < 0.001), and self-esteem was positively associated with close friend support (β = 0.324, *p* < 0.001). The specific indirect effect through self-esteem was *B* = 0.113, with a bootstrap 95% CI of [0.088, 0.138].

This pattern aligns with sociometer theory and related perspectives, which emphasize that perceived acceptance, respect, and relational value in salient social contexts are reflected in self-evaluations and may shape relationship engagement (Leary and Baumeister, 2000). Empirically, school connectedness and positive school experiences have been associated with self-esteem across adolescence (Aldridge and McChesney, 2018), and longitudinal evidence suggests that school experiences may be prospectively associated with later self-esteem (Varner et al., 2025). Although broader interpersonal functioning and relationship outcomes are not identical to close friend support, meta-analytic and longitudinal evidence indicates that self-esteem is linked to interpersonal functioning and relationship outcomes over time (Cameron and Granger, 2019; Harris and Orth, 2020). Within school peer ecologies, negative acceptance cues such as exclusion and rejection have been associated with lower self-esteem (Danneel et al., 2019), and social exclusion has been linked to adjustment outcomes through self-esteem and related psychological processes (Arslan, 2019). Taken together, the present findings are consistent with a statistical indirect association in which perceived school climate is related to close friend support, in part, through adolescents' self-esteem, while acknowledging that directionality and causal ordering cannot be established in the current design.

### 5.4 Sequential indirect association of basic psychological needs satisfaction in exercise and self-esteem

The findings were consistent with Hypothesis 4, indicating a statistically significant sequential indirect association between perceived school climate and close friend support through basic psychological needs satisfaction in exercise and self-esteem. The bootstrap estimate for the sequential indirect association (perceived school climate → basic psychological needs satisfaction in exercise → self-esteem → close friend support) was *B* = 0.046, with a bootstrap 95% CI of [0.035, 0.058]. At the path level, perceived school climate was positively associated with basic psychological needs satisfaction in exercise (β = 0.434, *p* < 0.001), basic psychological needs satisfaction in exercise was positively associated with self-esteem (β = 0.336, *p* < 0.001), and self-esteem was positively associated with close friend support (β = 0.324, *p* < 0.001).

This pattern is consistent with a theory-informed interpretation based on self-determination theory and sociometer theory: greater satisfaction of autonomy, competence, and relatedness needs in exercise settings may be associated with salient competence- and connection-related cues and more positive self-evaluations, which may in turn be related to constructive relationship engagement and supportive exchanges with close friends. Prior work has reported links between supportive motivational climates, need satisfaction, and indicators of positive development (Chen et al., 2020; Zheng et al., 2023), and evidence also supports positive associations between physical activity involvement and self-esteem (Liu et al., 2015; Shang et al., 2021; Laurier et al., 2024). Nonetheless, because the data were cross-sectional and the indirect-effect model was tested within a regression framework using observed variables, the sequential indirect association should be interpreted as a statistical decomposition consistent with the hypothesized order rather than evidence of temporal ordering or causal mediation. Alternative explanations should also be considered. For example, close friend support may contribute to adolescents' self-esteem rather than only being an outcome associated with it, and students with higher self-esteem may perceive their school climate more positively. In addition, unmeasured factors such as family support, academic stress, psychological distress, pre-existing friendship quality, peer network characteristics, and exercise participation characteristics may also be related to the observed associations. Therefore, the present model should be regarded as a theory-informed statistical model rather than evidence that the variables unfold in this temporal order. Future work using multi-wave longitudinal, cross-lagged, intervention, and multilevel designs will be important for evaluating temporal ordering and testing competing models more directly.

## 6 Practical implications

This study contributes to understanding correlates of close friend support in adolescence and offers practice-relevant considerations for aligning school-based mental health efforts with physical education. The findings suggest that students who perceive a more positive school climate also report higher close friend support, and that this association is statistically consistent with a sequential indirect association involving greater basic psychological needs satisfaction in exercise and higher self-esteem. Because the evidence was cross-sectional, the implications below should be interpreted as potential leverage points rather than causal prescriptions. Even so, the findings point to two potential areas that schools may consider addressing in parallel: improving students' day-to-day experiences of the school environment and shaping exercise contexts that are more likely to support autonomy, competence, and relatedness.

At the student-support level, schools may help students engage in exercise experiences that are compatible with autonomy, competence, relatedness, and supportive peer exchanges. Potentially useful strategies include (1) encouraging sustainable activity choices that students are willing to continue over time, which may support autonomy and personal choice; (2) setting attainable, improvement-focused goals for skills or fitness and using simple tracking and process-focused feedback to support competence, rather than relying primarily on social comparison; (3) participating in cooperative exercise settings, such as small-group training, coordinated team activities, or school clubs, to practice supportive communication, reciprocity, and conflict repair, which may provide opportunities for relatedness experiences relevant to supportive close-friend interactions; and (4) engaging in positive feedback and mutual help within peer interactions while seeking timely support from teachers, caregivers, or school mental health staff when exclusion or conflict occurs, which may help students access support during social difficulties.

At the school level, practice-relevant considerations can be organized around a high support-high structure approach. In Chinese senior high schools, where students often learn within relatively stable class groups and face strong academic demands, school-level practices may be especially relevant for shaping predictable, respectful, and supportive peer-interaction contexts. Structurally, schools may help reduce uncertainty in peer interactions by maintaining clear, fair, and consistently enforced rules, paired with transparent procedures for addressing bullying and exclusion. Relationally, schools may support students' sense of safety and inclusion by strengthening teacher support, encouraging respectful communication norms, and creating routine opportunities for positive peer connections. Within physical education and extracurricular exercise, schools may consider autonomy-supportive and mastery-oriented practices by offering meaningful choices, designing tasks with attainable challenges, providing process-focused feedback, and embedding cooperative goals, which together may support students' autonomy, competence, and relatedness during exercise. Finally, schools may coordinate physical education, classroom-based activities, and mental health programming by implementing peer-help initiatives and components focused on supportive close-friend interactions, alongside early identification and timely responses to bullying and exclusion. Coordinating school climate improvement with need-supportive exercise experiences and support for students' self-evaluative resources may help create conditions associated with higher perceived close friend support.

## 7 Limitations and future directions

This study examined whether basic psychological needs satisfaction in exercise and self-esteem statistically accounted for the association between students' perceived school climate and close friend support. Several limitations should be considered when interpreting the findings. These limitations also point to priorities for future research in measurement, study design, covariate adjustment, clustering, model testing, and generalizability.

### 7.1 Limitations of measurement methods

All study constructs were assessed at one time point using student self-report measures, which limits the ability to rule out shared method variance and response-style bias. Although a statistical screen for common method variance was conducted, single-source data may still inflate observed associations. In addition, although responses were screened for completion time, missingness, and obvious logical inconsistencies, the survey did not include formal attention-check items or systematic straight-lining detection. Self-report measures also cannot capture objective features of the school environment, instructional practices in physical education, or the structure of peer networks, limiting comparisons between perceived support and observed support or acceptance.

Two measurement-related issues warrant attention. First, perceived school climate was operationalized using the school-climate component of the SCASIM-St15, and school identification was not modeled as a distinct construct. Second, response-format adaptations used to harmonize scales may limit direct comparability with studies using the original formats. Future work could strengthen measurement by incorporating additional sources (e.g., teacher ratings, classroom observations, administrative records, peer nominations/peer reports, and social network indicators), applying more stringent approaches to common method variance assessment (e.g., marker variables or latent method factors), incorporating more rigorous careless-response screening procedures, and testing measurement invariance across sex and grade level.

### 7.2 Limitations in study design and causal inference

Because the study used a cross-sectional survey design, the results describe patterns of association and do not establish temporal ordering or causal direction. Accordingly, the sequential indirect association should be interpreted as a statistical decomposition consistent with the hypothesized order rather than evidence of temporal ordering or causal mediation. The design also cannot rule out reverse or reciprocal associations, particularly between self-esteem and close friend support, nor can it definitively determine whether basic psychological needs satisfaction in exercise functions as an antecedent, consequence, or reciprocal correlate within the proposed sequence. Future research should address these issues by using multi-wave longitudinal designs, ideally with at least three waves to evaluate temporal ordering, applying cross-lagged or longitudinal mediation models, testing interventions that target autonomy-supportive physical education and/or broader school-climate improvement using rigorous experimental or quasi-experimental designs, and adopting intensive longitudinal approaches, such as experience sampling, to examine shorter-term dynamics among exercise experiences, self-evaluations, and close friend support.

### 7.3 Limited adjustment for confounding factors

The analytic models controlled for sex and grade level only. Although these covariates were relevant to the study variables, other important individual, family, academic, psychological, peer-network, and exercise-related factors were not available in the present dataset and therefore could not be included as covariates. In particular, detailed indicators of socioeconomic status, such as parental education, parental occupation, or household income, as well as family support, academic stress, psychological distress, pre-existing friendship quality, peer network size and stability, and the type, frequency, and intensity of exercise participation were not collected in the present survey.

These factors may be related to students' perceptions of school climate, exercise experiences, self-evaluations, and close friend support. Therefore, the present findings should be interpreted as covariate-adjusted statistical associations rather than fully confounding-adjusted estimates or evidence of a highly specific explanatory pathway. Future studies should incorporate broader covariate sets more systematically and conduct robustness checks, such as alternative covariate sets and sensitivity analyses. Where feasible, multi-informant data and analytic strategies that strengthen confounding control would improve interpretability.

### 7.4 Model robustness and hypothesis testing

Indirect and sequential indirect associations were estimated within a regression framework using Hayes' PROCESS, and confirmatory factor analysis was used to evaluate measurement structure; however, competing theoretical models were not systematically compared within a full structural equation modeling framework. As a result, the current evidence is less informative regarding alternative explanations such as parallel indirect associations, alternative sequential orders, plausible reverse paths, or interaction effects. In addition, the study used observed mean scores rather than latent variables in the indirect-effect tests, which does not explicitly model measurement error. Because students were sampled within classes and schools, the primary analyses were conducted at the individual-student level and did not explicitly account for possible non-independence within classes or schools. This may have affected the precision of standard errors and significance tests. Future work should therefore retain and use school- and class-level information where feasible and consider analytic approaches that account for clustering, such as cluster-robust standard errors, multilevel modeling, or multilevel structural equation modeling. Future research can further strengthen model testing by comparing competing models using structural equation modeling with latent variables, evaluating global fit and information criteria, and conducting robustness checks using alternative estimators and resampling strategies.

### 7.5 Sample and generalizability

Participants were senior high school students from Shihezi, Xinjiang, China. Regional cultural context, school systems, and resource conditions may be distinctive; therefore, generalization should be made cautiously. Perceptions of school climate, experiences in physical education, and close friend support are context-dependent, and both construct levels and association strengths may vary across regions, school types, and management practices. In addition, Chinese senior high schools may differ in academic pressure, class organization, physical education resources, and school management practices, all of which may influence students' perceptions of school climate, exercise experiences, and close friend support. Replication across provinces, urban and rural contexts, diverse school types, and different grade levels-ideally using stratified multi-school sampling and multilevel modeling-would help evaluate the stability and generalizability of the proposed pattern.

### 7.6 Future research directions and applications

Building on the present framework, future work can extend this line of research in several directions: (1) decomposing basic psychological needs satisfaction in exercise into autonomy, competence, and relatedness to test their differential contributions and identify which need is most central in the sequential indirect association; (2) testing boundary conditions by examining moderators such as sex, grade level, type, frequency, and intensity of exercise participation, and experiences of peer exclusion to clarify for whom and under what conditions the proposed pattern is most likely to hold; (3) adopting multi-informant, multi-wave designs that integrate peer nominations/ratings and school-level indicators, applying cross-lagged or longitudinal mediation models, and using multilevel modeling to distinguish school-level structure from individual psychological processes; and (4) developing and evaluating theory-informed intervention modules that combine supportive school governance, autonomy-supportive physical education, and peer-help components, while assessing the durability, scalability, and potential effects of these approaches on exercise-related need satisfaction, self-esteem, and close friend support. Future studies should also examine whether the proposed associations differ across cultural, regional, and school-policy contexts. In addition, future studies may explicitly model school identification alongside perceived school climate to clarify their distinct roles and test whether the proposed associations differ when identification is treated as a separate construct.

## 8 Conclusion

In a sample of Chinese high school students, this study examined the association between students' perceived school climate and close friend support and evaluated whether basic psychological needs satisfaction in exercise and self-esteem statistically accounted for this association. The findings indicated that a more positive perceived school climate was associated with greater close friend support. This association included a remaining direct association and statistical indirect associations through basic psychological needs satisfaction in exercise and self-esteem, including a sequential indirect association involving both variables. Specifically, a more positive perceived school climate was associated with higher basic psychological needs satisfaction in exercise (autonomy, competence, and relatedness), which was associated with higher self-esteem and, in turn, with greater close friend support. From an adolescent developmental perspective, these findings integrate a school-context factor, an exercise-related motivational experience, and a self-evaluative resource within a single framework for understanding close friend support. They also point to practice-relevant considerations for improving students' day-to-day school climate experiences, supporting need-supportive exercise contexts, and strengthening self-evaluative resources in ways that may help create conditions associated with higher perceived close friend support.

Because the data were self-reported, collected at a single time point, and adjusted only for sex and grade level, the findings should be interpreted as covariate-adjusted statistical associations rather than evidence of temporal ordering or causal mediation. Future research should consider using multi-wave longitudinal designs and/or intervention studies and, where feasible, combine student reports with teacher ratings, peer nominations, and school-level indicators. Multilevel approaches across grade levels, school types, regions, and cultural contexts would further clarify the stability and generalizability of the proposed sequential indirect association.

## Data Availability

The original contributions presented in the study are included in the article/Supplementary material; further inquiries can be directed to the corresponding author.

## Supplementary material

Supplementary Table 1 Anonymized questionnaire response dataset used in the analyses, including coded demographic variables and item-level responses for the study scales.
